# Dr. Thomas Hughes Edmunds

**Published:** 1858-04

**Authors:** 


Dr. Thomas Hughes Edmunds was the third son of Richard and Lydia H. Edmunds, and was born in Cape May county, New Jersey, August 16th, 1818.

Early in life he chose the profession of dentistry, and was placed by his parents, when in the 18th year of his age, with Dr. Mathew Foster, in Arch street, Philadelphia. In the spring of 1838, having devoted himself assiduously to the study of his profession, he settled in Baltimore, Maryland, and commenced practice. In 1840, he was married; and in 1848, being in delicate health, and advised to seek a country residence, he removed to Essex county, Virginia, where he remained practicing in that and adjacent counties about eighteen months; but finding a country practice too laborious, and requiring his too frequent absence from his family, he again removed to a city, choosing Richmond, Virginia, as a location. He soon established himself in a fair practice, which continued until suddenly snatched from his family by death. He, however, acquired not more than a competency, being devoid of that characteristic which is regarded by some as the greatest of worldly wisdom—the peculiar faculty of hoarding earthly treasure. He was, on the contrary, liberal and generous *to a fault.* A pressure of business at the time, which induced him to make extra exertions, brought on the typhoid fever; and after seven weeks of intense suffering, he died on the 14th of January, 1857, in the 39th year of his age, causing the first blank in a family of twelve children, all grown.

Dr. E. had many warm friends—numbers of his patients became such from only a professional acquaintance. His manners in his office were exceedingly suitable to a dentist, combining firmness and consideration, with gentleness, patience and forbearance—qualities essential to success in pleasing; while his skill was considered inferior to none in his profession. He availed himself of all the real improvements, but had a great repugnance to humbugs. When the use of chloroform in surgical dentistry was called for, he was always very cautious; and though having administered it about eight hundred and sixty times, without accident or injury, he never preferred it. Dr. E. was one of the original signers of a petition to the Legislature to charter the first Dental College in the state of Maryland; he was then practicing in Hanover street, Baltimore.

His early death has caused a widow and five children to mourn, when they most needed his counsel, direction and support, and for whose sake, mainly, he expressed himself, on his death bed, anxious to live.

The writer of this brief and imperfect tribute to the memory of one he loved, having known him for nearly seven years previous to his death, and having been connected with him in business, feels that by his death he has lost a valued friend, and the dental profession an honorable member. S.

RICHMOND, *October* 20*th*, 1857.

